# Integrative biology of persister cell formation: molecular circuitry, phenotypic diversification and fitness effects

**DOI:** 10.1098/rsif.2022.0129

**Published:** 2022-09-14

**Authors:** Alicia Berkvens, Priyanka Chauhan, Frank J. Bruggeman

**Affiliations:** Systems Biology Lab, AIMMS, VU University, De Boelelaan 1087, 1081 HV Amsterdam, The Netherlands

**Keywords:** systems biology, persister cell microbiology, mathematical models, microbial fitness and bet hedging

## Abstract

Microbial populations often contain persister cells, which reduce the extinction risk upon sudden stresses. Persister cell formation is deeply intertwined with physiology. Due to this complexity, it cannot be satisfactorily understood by focusing only on mechanistic, physiological or evolutionary aspects. In this review, we take an integrative biology perspective to identify common principles of persister cell formation, which might be applicable across evolutionary-distinct microbes. Persister cells probably evolved to cope with a fundamental trade-off between cellular stress and growth tasks, as any biosynthetic resource investment in growth-supporting proteins is at the expense of stress tasks and vice versa. Natural selection probably favours persister cell subpopulation formation over a single-phenotype strategy, where each cell is prepared for growth and stress to a suboptimal extent, since persister cells can withstand harsher environments and their coexistence with growing cells leads to a higher fitness. The formation of coexisting phenotypes requires bistable molecular circuitry. Bistability probably emerges from growth-modulated, positive feedback loops in the cell's growth versus stress control network, involving interactions between sigma factors, guanosine pentaphosphate and toxin–antitoxin (TA) systems. We conclude that persister cell formation is most likely a response to a sudden reduction in growth rate, which can be achieved by antibiotic addition, nutrient starvation, sudden stresses, nutrient transitions or activation of a TA system.

## 1. Introduction

### 1.1. Persister cells as insurance policies against extinction

It appears that many, if not all, bacterial species generate subpopulations of non-growing and stress-tolerant persister cells, as perseverant ‘seed banks’ that enable a species to recover and repopulate its niche after catastrophic events [1]. Persister cell formation is probably just one aspect of a more general bacterial strategy to cope with dynamic, sometimes hostile, environments.

### 1.2. The discovery of persister cells

In 1944, Joseph Bigger discovered persister cells of *Staphylococcus* [2]. When he plated cells and exposed them to penicillin, he realized that some survived treatment. Since growth did not occur in presence of penicillin, he concluded that the surviving fraction had to be genetically identical to the killed ones. The surviving cells were therefore not resistant to the antibiotic, but tolerant instead, and Bigger named them persister cells.

### 1.3. A definition of persister cells

Following Harms *et al*. [3], we define persister cells ‘as a subpopulation of cells in a bacterial population that exhibits tolerance to antibiotics and other environmental stress conditions because of a phenotypic transition into a dormant state in which the cellular processes commonly poisoned by bactericidal antibiotics are inactive’. Thus, persister cells hardly grow, or not at all, and somehow coexist with genetically identical growing cells. They are formed in response to environmental changes including stresses, antibiotics or nutrient transitions, or by chance [4,5].

### 1.4. Many bacterial species display persister cell formation

Many bacterial species have proved to manifest subpopulations of persister cells [6,7], including human pathogens like *Pseudomonas aeruginosa* and *Mycobacterium tuberculosis,* as well as *Lactococcus lactis* [8] and *Escherichia coli* [5]. The fraction of persister cells in a population appears species and environment dependent. The physiological state of a persister cell has been studied extensively, pointing to various origins [9,–12], but a consensus mechanism remains to be identified. Persister cells are believed to be in a distinct phenotypic state [13,14] with the expression of toxin–antitoxin (TA) systems and often share similarities with starvation phase cells [14].

### 1.5. Antibiotic tolerance facilitates evolution of resistance

Persister cells may have remarkably wide evolutionary implications. For instance, antibiotic-tolerant persister cells do not only pose a health risk—because they can transiently survive antibiotic treatment and re-establish a growing population of cells afterwards. They have also been associated with accelerating the evolution of antibiotic resistance [15–17], which is an even greater threat. Persister cells provide a genetic reservoir of resistance mutations (since they survive treatment) and allow for the evolution of mutants that are antibiotic resistant (e.g. multidrug-resistant strains [15,17,18]). This example shows that persister cells may have wide implications, not only for medical microbiology, but also for microbial ecology, since dormant cells are widespread in nature [1].

### 1.6. An integrative evolutionary, molecular and physiological perspective

Although the existence of persister cells has been known for almost 80 years, how they exactly form, and how this relates to microbial physiology, remains a topic of discussion. Over the years, key molecules have been implicated in persister cell formation, of which some have a central metabolic role, but a true understanding of their roles remains elusive. For instance, ATP reduction [19], guanosine pentaphosphate (ppGpp) rise [20] and toxin excess over antitoxins [3] have all been shown to play a role (mostly in *E. coli*), but none of them appears necessary [20–22]. Persister cell formation seems to be a systemic effect that emerges upon qualitatively different cellular perturbations, via multiple routes.

Since persister cell formation is widespread across bacterial species, and its underlying mechanisms appear so conserved [3,23], it is reasonable to consider an overarching conceptual framework that offers an evolutionary perspective on the fitness merits of persister formation, with which we can rationalize its physiological and molecular aspects.

Also, since persister cell formation is probably intertwined with the regulation of cellular metabolism [24], exercising an evolutionary and physiological perspective on persister formation might prove insightful. Such an overarching framework is currently lacking. The existing theoretical studies often take a phenomenological approach, without considering the relation of underlying molecular circuitry causing the phenotypic switching to the cell's physiology; or they take a purely non-mechanistic fitness approach [25,26]. While the experimental studies often focus on a single causal mechanism, at particular conditions, without relating this back to whole-cell regulation mechanisms and long-term fitness effects.

**Figure 1.**
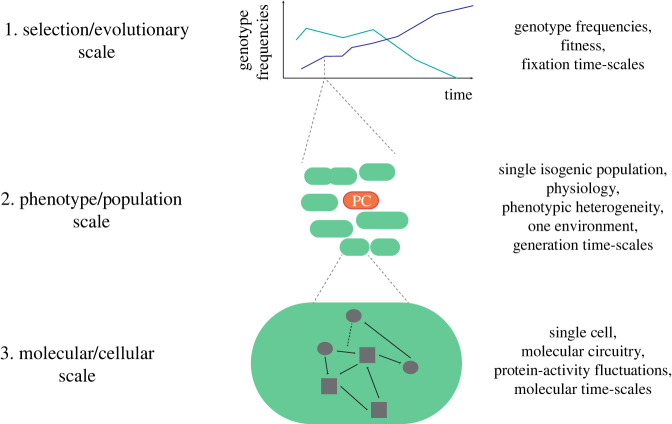
The middle-way approach to understand persister cell formation in an integrative manner. An overview of the three different scales (evolutionary, physiological and molecular) that we aim to integrate to achieve an improved molecular–physiological–evolutionary understanding of persister cell formation.

We argue that we need a ‘middle-way approach’ [27] that relates changes in evolutionarily meaningful, physiological (phenomenological) parameters (such as phenotypic switching rates and growth rates) to the underlying molecular properties and circuitry, in the context of a cell that is undergoing a constrained adaptation to its environment. This approach should contribute a more integrative molecular–physiological–evolutionary understanding, which indeed appears possible as illustrated by recent ‘systems’ advances in microbial physiology [28]. In this review, we aim to contribute to such an integrative perspective by discussing and relating important persister cell hallmarks of the molecular, physiological and evolutionary ‘scales’ (figure 1).

## 2. A growth versus stress trade-off may underlie persister cell formation in bacterial populations

### 2.1. Limited biosynthetic resources and protein expression trade-offs

Shortly after the introduction of the concept of balanced growth by Campbell in 1957 [29], which effectively turned microbiology into a quantitative science [30,31], Maaløe and Kjeldgaard [32,33] concluded in 1966 that the bacterial growth rate is the outcome of regulatory systems that allocate limited biosynthetic resources over genes, i.e. RNA polymerases (RNAP), nucleic acids, energy equivalents, ribosomes and amino acid-loaded tRNAs (see also [34]). They hypothesized that optimal allocation of these resources would maximize growth rate and would often be the outcome of natural selection [34–37]. This conceptual framework has proven remarkably powerful in the last two decades [28,38–40].

A direct corollary of this view is that the growth rate of cells will decrease when they express unneeded proteins—Monod called them gratuitous proteins—as their biosynthesis will be at the expense of growth-promoting proteins, which is indeed what is experimentally found [39] (see electronic supplementary material, A).

### 2.2. The growth versus stress trade-off results from the allocation of finite-transcriptional resources and its regulation

In many bacteria, ppGpp and sigma factors are the key regulators of the allocation of a cell's finite-biosynthetic resources over growth (metabolic and ribosomal) and stress operons [41]. For instance, in *E. coli,* during amino acid shortage and ribosome excess, ppGpp synthesizing enzymes (members of the RelA/SpoT homologue (RSH) family) are activated [42]. When uncharged tRNA binds to the ribosomal A-site RelA initiates ppGpp synthesis, thereby acting as a sensing mechanism for imbalances in ribosomal versus metabolic investment [37,43]. ppGpp then diverts RNAP (complexed to DksA and *σ*^70^ [33]) away from ribosomal operons to catabolic operons such that amino acid biosynthesis is increased. When nutrients are scarce, ppGpp diverts RNAP also to stress operons (then *σ*^S^ is involved) [44]. Therefore, ppGpp has a direct influence on fitness by both impacting cellular growth rate and stress tolerance [37]. Furthermore, ppGpp has been implicated in persister cell formation [24].

The alarmone ppGpp is also a major determinant of the competition for RNAP by sigma factors in *E. coli*. Sigma factors are necessary subunits of RNAP and therefore associated with growth or stress-related gene expression [45,46]. In many bacteria, transcription initiation requires a dedicated sigma factor, which binds to RNAP to initiate transcription, after which the sigma factor releases, and the RNAP moves along the gene to make its mRNA product [47]. *Escherichia coli* has seven sigma factors [48], each responsible for transcription of different classes of genes: growth (housekeeping) (*σ*^70^), nitrogen-limitation metabolism (*σ*^N^), flagellar biosynthesis (*σ*^F^), heat shock (*σ*^H^), envelope stress (*σ*^E^), starvation phase (*σ*^S^; RpoS) and FecI (ferric citrate transport).

The fraction of RNAP (a limited biosynthetic resource) allocated to a particular cellular process depends therefore on the outcome of the competition by sigma factors for RNAP binding. The concentration of sigma factors is regulated and condition dependent and co-determines the allocation of biosynthetic resources over different cellular tasks such as growth versus stress.

Thus, whenever a cell is confronted with a stress and has increased *σ*^H^, *σ*^E^ and *σ*^S^ concentrations, it will have an increased demand for expression of stress-associated genes. The resulting RNAP allocation to stress sigma factors is at the expense of RNAPs available for growth-associated sigma factors (*σ*^70^ and *σ*^N^), and the cell will generally grow slower. This mechanism implies a trade-off between growth and stress, as suggested by Nyström [49] and Ferenci [50], implying that cells cannot simultaneously grow fast and be highly stress tolerant. Indeed, it has been shown experimentally that slow-growing *E. coli* cells are more stress-tolerant than fast-growing cells [51–53], and that fast-growing cells adapt slower to new conditions than slow-growing cells [54], which apparently have enhanced anticipatory protein expression [53].

Thus, a single bacterial cell cannot invest in fast growth and high stress tolerance simultaneously. However, if we look at it from a population level, a bacterial species can still accomplish this by the formation of two distinct phenotypes. This two-phenotype strategy will be discussed in the following section, in the light of its fitness consequences.

## 3. Fitness aspects of persister cell formation

### 3.1. Why two phenotypes, one growing and the other stress-tolerant, rather than a single phenotype?

Persister cell formation is probably an adaptation to a changing environment that can rapidly turn into an extinction-threatening condition [55]. However, when a two distinct phenotype strategy is more favourable than one based on a single phenotype, investing in both growth and stress tolerance, is not immediately obvious. (Note that we consider a two-phenotype strategy, but that in reality the population may consist of more than two phenotypes.)

The growth versus stress trade-off outlined above indicates that fast growth and high stress tolerance are probably two opposing strategies. One possible way to cope with this trade-off might be phenotypic diversification, as this enables the coexistence of fast-growing and stress-tolerant cells. However, since the stress-tolerant phenotype hardly grows, the net growth rate of the associated genotype reduces with the size of the stress-tolerant subpopulation (linearly as we will see below). Since the growing, stress-tolerant phenotype of the single-phenotype strategy also has a reduced growth rate, one can wonder under which conditions the phenotypic-diversifying genotype is fitter than the single-phenotype genotype.

### 3.2. Long-term fitness and immediate fitness costs of persister formation

**Figure 2.**
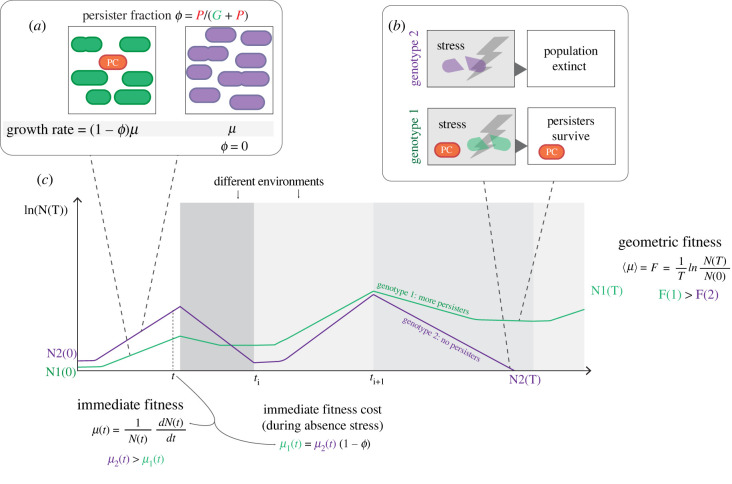
Graphical description of differences in a genotype able to make persisters and one that is not. (*a*) A visual representation of a genotype I (green) which is able to form persister cell (PC) (orange), and genotype 2 (purple) which is not. The fraction of persister (*ϕ*) in genotype 2 is therefore always 0, which means that its growth rate during unstressed conditions is higher than that of genotype 1 with *ϕ* greater than 0. However, a severe stressful condition (dark grey block) would be able to kill all cells of genotype 2 (*b,c*), leading to extinction. Different shades of grey represent different environmental conditions. See electronic supplementary material, B–D and G for a coarse-grained mathematical description of short- and long-term fitness effects of forming a persister fraction as depicted here.

In evolutionary biology, the accepted definition of long-term fitness of a genotype, here denoted by *F*, over a period of dynamic conditions with total duration *T*, is the logarithm of the fold-change in cell abundance divided by *T* [56]. This measure equals the average (specific or per capita) growth rate of this genotype, denoted by 〈*µ*〉, during *T* (electronic supplementary material, B). Thus, the fittest genotype grows the most in cell number; it has the largest abundance increase factor, despite possible losses in abundance due to cell death during stress conditions (figure 2). We define the short-term fitness in the same way, but apply it to time durations much smaller than *T*, typically of the order of the generation time. As a consequence, short-term fitness equals the immediate (specific or per capita) growth rate, which we shall denote by *μ* (figure 2).

One strategy for a genotype to maximize the long-term fitness 〈*µ*〉 is to always maximize its short-term fitness and, therefore, always grow as fast as maximally possible. The risk of this strategy is extinction when a sudden stress event occurs. Microbial species that employ a two-phenotype strategy and form stress-tolerant, non-growing persisters can overcome this risk, but have to pay a fitness cost. In the electronic supplementary material, G, we show that the immediate growth rate of the persister-forming genotype reduces from *μ* to (1 − *ϕ*)*μ* with *ϕ* as the persister fraction, defined as the number of persister cells divided by the total number of cells (figure 2).

### 3.3. The evolutionarily optimal persister fraction equals the probability for an extinction event

When a bacterial species grows at a constant growth rate for a long enough time, the persister fraction eventually becomes constant and reaches a steady state value (see electronic supplementary material, D). Since the presence of persister cells reduces the immediate growth rate, a natural question to ask is what the persister fraction is that maximizes long-term fitness.

With a simple model (see electronic supplementary material, E), it can be shown that the optimal phenotype fraction equals the chance of an extinction-threatening condition [57]. Thus, the phenotype fraction can be very low if such a condition is a rare event. This makes the fitness cost also very low, effectively making the persister-forming strategy a very competitive strategy under such circumstances.

### 3.4. Optimal phenotype-switching rates maximizing long-term fitness

The steady state persister fraction is in the simplest case dependent on the growth rate and the phenotype-switching parameters (electronic supplementary material, D). Therefore, setting the persister fraction to its optimal value does not also set the phenotype-switching parameters. What determines the optimal values of the switching parameters, which maximize long-term fitness, has been addressed in several papers [25,26].

Intuitively, the most optimal case would be for a single cell to remain a persister for just as long as the stressful condition lasts such that it switches to the growing state when a favourable condition starts. In electronic supplementary material, F, we outline the results of Gáal *et al*. [26]. They found that the optimal switching rates equal $k\approx 1/\tau +1/(\Delta \mu )\tau^{2}$, when the durations of the growth and stress environment equals *τ* and Δ*μ* equals the growth rate difference between the two conditions, which indicates that the phenotype-switching rates should match the environment switching rates, in agreement with the intuition and evolution experiments [58,59].

### 3.5. Why phenotypic diversification, instead of adaptation of the mean cell?

In principle, an alternative, *one-phenotype* strategy could exist where each cell grows and is also prepared for stress conditions, such that all cells grow and survive sudden stress conditions. This strategy's fitness cost, quantified as the reduction of the immediate growth rate, relates to the protein investment into stress at the expense of growth-supporting proteins. Experiments and theory indicate that the growth rate now reduces from *μ* to (1 − *f_s_*)*μ* (see electronic supplementary material, C and figure 2), with *f_s_* as the mass fraction of preparatory stress proteins. Note that this relation reflects the growth rate versus stress trade-off, which was introduced above.

Thus, the persister-forming, two-phenotype strategy has a higher short-term growth rate if *ϕ* < *f_s_*. Since persister cells do not grow at all and might therefore have global changes in protein expression that are larger than for cells that also grow, it appears that phenotypic diversification is nearly always the best strategy when stress conditions are infrequent. When conditions change rapidly between stress and growing conditions then a bifunctional phenotype, growing and stress tolerant might be selected for.

### 3.6. Are the evolutionary insights based on a too simplified and phenomenological model?

The purpose of the evolutionary analysis is understanding of optimal parametrizations of the basic persister formation mechanism, which constitutes a prediction of the expected biological outcome, and suggests null hypotheses when we rationalize measured parametrizations of bacterial species. The problem is, however, that the evolutionary analysis was based on a phenomenological model. To what extent this simple model approximates the phenotypic consequences of the underlying molecular circuits responsible for persister formation and resuscitation remains poorly understood. This also has not been addressed in the literature to date. We have to cross scales from physiology to molecular biology to understand whether the underlying molecular circuits really give rise to this emergent population dynamics.

## 4. Molecular biology of persister cell formation

A key aspect of persister biology is that a single bacterial species (a genotype) is capable of generating two genetically identical, but physiologically distinct, phenotypes that coexist at a single environmental condition, one adapted to the current environmental state and the other to a possible future environmental state. For instance, under a growth-supporting condition the abundant phenotype grows and the persister does not (or hardly) and is stress prepared. Alternatively, in the stationary phase or in biofilms, the persister phenotype(s) coexists with non-persister cells and are both not growing. In any case, the coexistence of two phenotypes in a clonal population, and the switching from one phenotype to the other, needs to be explained, as it is not a general outcome of all molecular circuits.

### 4.1. Bistability in molecular circuitry underlying persister formation

A first requirement for the coexistence of two isogenic phenotypes at a constant condition is bistability of the underlying molecular circuit(s). Bistability is well understood and a property of particular, often remarkably simple, molecular circuits, generally involving (a) net positive feedback(s) [60,61]. It gives rise to the counterintuitive phenomenon that genetically identical cells growing under the same condition end up in a different stable, phenotypic state due to a chance event, e.g. due to fluctuating expression levels of transcription factors. For instance, because of a transcription factor concentration exceeding a critical threshold, above which a self-perpetuating (net) positive feedback loop is activated that further increases this concentration, such that the cell moves away from its initial state, whereas the cell with a transcription factor concentration below the threshold remains in its low-concentration state. Positive autoregulation of gene expression of a transcription factor can lead to this type of behaviour [60,61].

### 4.2. Phenotype switching is required for persister cell formation and resuscitation

Reversible phenotype switching is a second requirement for persister cell formation and their resuscitation. Reversible switching requires large enough spontaneous fluctuations of concentrations of regulatory molecules to allow the system to transit from one phenotypic state to the other (and back); so, concentration fluctuations that pass the threshold concentration upwards or downwards [55].

Since concentration fluctuations are inherent properties of the molecular circuits inside cells [62], all bistable systems fluctuate and can, in principle, display stochastic reversible switching [55]. However, those random switches may be very rare. The switching rate constants in the population dynamics model (which we considered in the fitness analysis above) represent the bistability-associated rates of switching from growing to persister cells and back. These model rate constants are therefore phenomenological, as they correspond to the frequency of large-enough fluctuations that cause the molecular circuit to transit to the other steady state. Thus, the switching rate constants in fact represent a complex (systemic) property of the underlying bistable molecular network, which can generally not be expressed (mathematically) in terms of kinetic properties of the molecular network. To understand better how bistability can arise in the molecular networks associated with persister cell formation, we will discuss a few of their key aspects. We will discuss this in the light of TA systems, ppGpp and sigma factors which have all been linked to the formation of persisters.

### 4.3. Bistability of toxin–antitoxin systems

**Figure 3.**
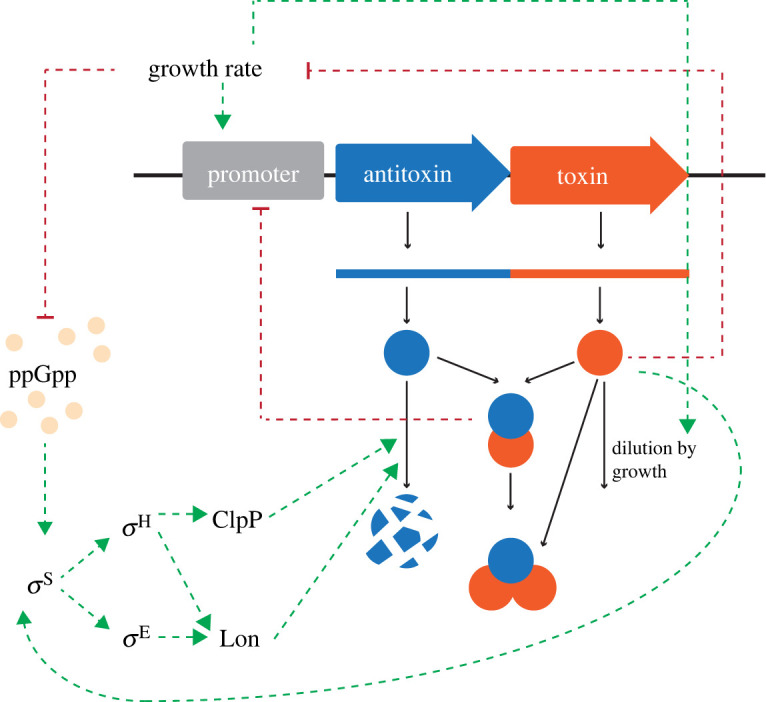
Mode of action of type II TA systems, which appears inherently bistable. A schematic overview of the mode of action of a type II TA system, which appears inherently bistable. It shows several interlocked, self-perpetuating positive feedbacks that are in principle capable of giving rise to bistability. All the sigma factor dependencies were identified in EcoCyc (www.ecocyc.org). Note that the influence of the toxin on *σ*^H^ and *σ*^E^ is toxin dependent and is exerted through *σ*^S^ [76]. Some toxins also directly influence protein synthesis. (Note that for ribosome targeting antibiotics, like tetracycline, the relation between growth rate and ppGpp is opposite from depicted here [43].)

TA systems are proposed to be linked to persister cells ever since the discovery that mutations in the toxin gene hipA were shown to cause high persister frequencies in *E. coli* [63]. Conversely, the deletion of single toxins can decrease persister cell fractions (e.g. of MqsR [64], TisB [65] and YafQ [66]). While the exact effects of TA systems on persister formation are not yet understood, and some researchers even question whether TA systems play a role at all [67], a role for TA systems has never been truly ruled out [68]. There still seems considerable evidence for a relation to persistence [69–72]. However, out of seven types of TA systems, how bistability arises is understood only for type II systems and only from mathematical models [3,61,73,74] that incorporate conditional cooperativity and growth-modulating positive feedback [75] (figure 3). (This mechanism regrettably lacks sufficient experimental proof.) In fact, the contribution of type II TA systems to persister cell formation in unstressed cells has recently been seriously challenged [68,77,78]. So, how TA systems lead to the occurrence of persister cells in the presence of growing cells, and how they are capable of switching back to growing cells, is not yet understood. We will propose some candidate mechanisms below.

### 4.4. Growth-modulated positive feedback

A growth-modulated feedback mechanism can bring about bistability, as was nicely experimentally illustrated by Tan *et al*. [75]. They showed that a system, which is composed of a protein that positively activates its own synthesis and inhibits growth by consuming biosynthetic resources that would otherwise benefit growth rate, as a toxin of a type II system does, can exist in two states. In one state, growth is fast and the protein is at a too low concentration, because of a too high dilution by growth, for it to inhibit growth (figure 3). In the other state, its concentration is high and the growth rate is low, due to inhibition. A critical concentration of protein now exists. Above it, the system moves to the growth-inhibited state and, below it, the cell grows and experiences only a small protein burden from inhibition (figure 3). This mechanism could function for TA systems that directly inhibit growth [3,23], such as theoretically shown for HipAB by Klumpp *et al*. [69] and Cataudella *et al*. [61].

### 4.5. Sigma factors, (p)ppGpp and toxins, in persister formation

We have already hinted at the importance for ppGpp and sigma factors in the regulation of growth rate versus stress preparedness and mentioned that high levels of ppGpp and upregulated *σ*^S^ are linked to the physiological state of a persister cell [14]. How could bistability arise in this case?

One possibility is that activation of *σ*^S^ leads to bistability, via a growth-modulated positive feedback loop. First, *σ*^S^ activation sequesters RNAP away from *σ*^70^, reducing the biosynthesis of metabolic enzymes and ribosomes, which reduces growth rate and leads to a ppGpp increase. This increase in ppGpp further activates *σ*^S^ synthesis and reduces synthesis of *σ*^70^ (figure 3). This is a net self-perpetuating feedback loop that may lead to persister formation in some cells, depending on their initial state, while other cells remain in the growing state—in the same manner as the growth-modulated positive feedback mechanism.

Moreover, rises in ppGpp and *σ*^S^ due to slow growth, similar to a stationary phase state [14], may lead to the induction of particular TA systems [19,70,79]. Several TA mechanisms have been shown to be connected to ppGpp. For instance, the toxin MazF is activated by ppGpp [80]. Since MazF is linked to ppGpp, its activation is linked to all growth rate-reducing perturbations (e.g. onset of starvation phase by nutrient limitation, nutrient transitions and additions of serine hydroxymate) that lead to a rise in ppGpp. If this activation continues long enough, *E. coli* transits to a starvation phase, via the induction of *σ*^S^ (e.g. [14]). MazF is activated by ppGpp via a route different from the recently questioned route proposed by Harms *et al*. for type II TA systems that involves polyphosphate accumulation and degradation of antitoxins by Lon [81]. Instead, the MazF p2 promoter is directly regulated by ppGpp [80].

Thus, ppGpp probably plays a central role in persister formation. However, Amato *et al*. [20] showed that *E. coli* forms persister cells during nutrient transitions, which involved ppGpp and cAMP. They also found that additional mechanisms were active as ppGpp and cAMP negative mutans also showed persister cell formation. These results suggest that alternative, ppGpp-bypassing mechanisms for persister formation exist. This might indicate that (some) TA systems, or a TA-independent mechanism, are responding to a growth rate reduction, independently of ppGpp. One such alternative route might be the induction of proteases, in particular those that degrade antitoxins, i.e. ClpAP, ClpXP and Lon protease [79], which are both indirectly under control of *σ*^S^ and induced at a low growth rate. The resulting network of molecular interactions has several interlocked, self-perpetuating positive feedbacks that are in principle capable of giving rise to the bistability associated with persister formation.

## 5. Back to physiology—when persisters form

### 5.1. Reversible phenotype switching remains elusive

Summarizing, there are several key molecular systems associated with persister formation in a growth rate-dependent manner such as ppGpp, TA systems and sigma factors, which seem capable of inducing bistability—independently or intertwined. Still, it remains elusive how they exactly cause reversible phenotypic switching, under which conditions, and how they synergize. Yet, somehow their persister formation effects are triggered by different conditions. Answering this question demands a more quantitative approach that determines the relative contributions of these alternative mechanisms to persister cell formation. We will now zoom out again and discuss persister formation at the physiological level, under stressed and growing conditions, to try to relate the previously discussed higher level fitness-based concepts to mechanisms at the molecular level. We shall take again a middle-way approach, representative of our integrative biology approach to persister formation.

### 5.2. Chance-based persister cell formation

The formation of persister cells in bacterial populations is believed to be the result of both chance-based and responsive mechanisms. During growth-supporting, stress-free conditions, persister cell formation has been suggested to be chance based and an example of bet-hedging [5]. The rationale is that at the level of a single cell, spontaneous and random fluctuations in gene expression induce fluctuations in protein expression that can ‘flip bistable switches', giving rise to a growing and persister cell subpopulation in an isogenic cell population. Evidence was obtained for this phenomenon by Balaban *et al*. [5]. That persister cell formation should occur under growing conditions is typically assumed in the field, but besides the above-mentioned (indirect) experiment evidence is lacking.

Some believe, however, that persister cell formation is purely a responsive phenomenon. For instance, it has been suggested that persister cells during fast growth of *E. coli* are a carry-over from a stationary-phase inoculum [82,83]. However, unlike *E. coli*, maintaining *Mycobacterium smegmatis* in exponential growth for four sequential growth passages (inoculum size constant) does not eliminate persister cells, as similar fractions of isoniazid- and ciprofloxacin-tolerant persister cells were found [22]. Since the persister fraction does not diminish, persister cells are continuously formed. Also, the absence of persister cells during exponential growth would leave the genotype unprotected against sudden hostile conditions.

Finally, pure chance-based formation of persister cells during growing conditions is perhaps often not evident, because the associated persister fractions are so low, of the order of one in a million cells [5]. According to the fitness theory (mentioned above), that fraction may reflect the probability for the occurrence of an extinction-threatening event. In electronic supplementary material, I, we explain how an unequal division of TA molecules could lead to chance-based persister formation.

### 5.3. Responsive persister cell formation

Persister cells can also form as a response to a stressful environmental change. Various stresses have been implicated in persister cell formation such as environmental stresses, such as heat shock, oxidative stress and antibiotic treatment [21,65], or physiological stresses that bacteria experiences during growth; for instance, during nutrient transitions or limitations [4,14,19,84], pH changes or antibiotics additions. The fitness risk of responsive persister cell formation is that it can be too slow such that too many cells are killed, due to the stress, before they have responded adequately. It appears that anticipatory persister formation (e.g. chance based) during stress-free conditions can protect against this. The fitness benefit of responsive mechanisms is, however, that the growth rate during stress-free conditions is not reduced, due to stress tolerance associated with protein synthesis, prior to the stress (as discussed above).

### 5.4. Is responsive persister formation a response to a sudden growth rate reduction?

**Figure 4.**
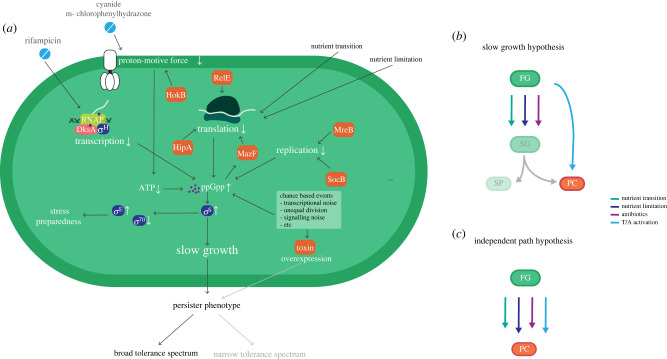
How different toxins and external stresses all reduce slow growth, providing a general trigger for persister cell formation. Different toxins (their antitoxin counterparts not visualized) and stresses (e.g. antibiotics) inhibit transcription, translation and replication through different mechanisms. Regardless of their causal mechanisms, these all lead to a reduction of cellular growth rate. Slow growth or growth halt seems a general stimulus for increasing the chance that an initially growing cell switches to a persister phenotype. Toxins are shown in orange, sigma factors in dark blue, antibiotics in light blue, DksA in pink, RNAP in green, mRNA in white, a ribosome in dark green and ppGpp in purple.

In figure 4, we illustrate several responsive and chance-based mechanisms related to induction of persister formation, highlighting the common effects of the plethora of persister-forming events. What antibiotic administration, nutrient transitions, chance-based toxin activation and all other events depicted in figure 4 have in common is that the growth rate suddenly drops. We postulate that persister cell formation is a responsive event to such a sudden growth rate reduction.

The reduction of protein production, e.g. by ribosome inhibition (tetracycline [21]) or toxin production [69], leads to persistence formation in *E. coli*. Thus, antibiotics and toxins that inhibit translation, and thereby stall growth, can induce persistence. Moreover, the inhibition of transcription with rifampicin, and of ATP synthesis with carbonyl cyanide *m*-chlorophenylhydrazone, enhanced the number of persister cells in an *E. coli* culture [21]. The subsequent reduction in growth rate is generally accompanied by a rise in the ppGpp concentration in *E. coli* [41], which causes the activation of *σ*^S^ and the onset of the generalized stress response [85]. It is tempting to speculate that this mechanism is also capable of inducing persister formation; thus, a metabolic route linked to *σ*^S^ [20], as discussed above*.* Similarly, the stationary phase of a bacterial culture, which is characterized by slow-growth and high-stress-tolerant physiology, harbours 10^3^ to 10^4^ times more persisters than exponentially growing cultures. This high frequency of persisters in this phase may probably be because of the involvement of *σ*^S^ during this phase, which is under control of ppGpp [86].

Another example, which strengthens the slow-growth hypothesis, is persister cell formation during nutrient transitions, a phenomenon observed in different studies [4,14,19,84]. A corollary of the growth versus stress trade-off is that *E. coli* is less capable of transiting to new nutrient conditions when it grows fast [53,54]. The growth rate of a cell decreases upon nutrient depletion. A cell then experiences a period of nutrient starvation, during which internal pools of key metabolites can deplete. This phase is probably accompanied by drops in energy-generation and biosynthesis rates (thus growth rate), reducing ATP and increasing ppGpp levels. This starvation period continues until a cell has adapted to the new nutrient condition—by appropriately adjusting its protein expression—and shows restored growth with replenished metabolite pools. But for some cells, this transition and starvation period might take too long, ppGpp may rise to such high levels that it induces a phenotypic switch that resembles a transition to a nutrient-starved state [14,87]. (Note that observations of subpopulations of cells after nutrient transitions may be dependent on the exact procedure by which cells were transited [87].)

In recent years, the ‘slow-growth hypothesis' (figure 4) has gained attention from different groups [11,14,88]. This hypothesis would explain why ppGpp, *σ*^S^, ATP and the induction of stationary phase have all been associated with persister cell formation, as these are all associated with a growth rate-reduction response. How such a slow-growth response could lead to phenotypic diversification in only a subpopulation of the cells remains an interesting open question.

### 5.5. Generalize or specialize your persister cells?

Although we propose that persister cell formation is probably triggered by a general physiological phenomenon—a sudden growth rate reduction—this does not imply that all the formed persister cells are identical with respect to their stress tolerance properties. One observation hinting that persister cells are heterogeneous is that the persister fraction often appears antibiotic dependent [65,89–91]. The fitness benefit of such heterogeneity is clear; bacteria that are often exposed to certain stresses are expected to have a large fraction of specialized persisters that are able to withstand that particular stress (this follows from the fitness theory), each with formation probabilities reflecting the likelihood of the stress they are tolerant for. Evidence for this exists [20,21], but has not been directly observed. This specialization of persister cells might follow from the observation that toxins have different ways to stall growth [23] and that different TA systems may become activated as part of the slow growth-rate response.

### 5.6. Heterogeneity of persister cells

Persister cells may turn out to be heterogeneous, presumably originating via diverse mechanistic routes and exhibiting varied tolerances towards different antibiotics. In this way, persistence provides bacterial cells an adaptive advantage for being prepared for several stresses. This may work via different TA systems, each given a unique phenotype with particular tolerance properties [3].

That persister cells are probably heterogeneous and exhibit a more complex phenotype than is sometimes realized, is also evident from the involvement of intracellular stress responses in persister cell formation [92]. The screening of knockout libraries for persister genes, and transcriptome analysis of isolated subpopulations of persister cell indicated that several persister formation mechanisms operate independently and in parallel [6]. These redundancies make stress tolerance heterogeneity among persister cells all the more likely. All of this is further substantiated by kill-curve results of the same cell population exposed to different antibiotics, which invariably show different persister fractions and cross-tolerance to antibiotics. This has been convincingly demonstrated by Kwan *et al.* [21]. They showed that cells pretreated with rifampicin lead to 59% survival on ciprofloxacin and 69% survival on ampicillin, whereas cells pretreated with tetracycline showed 47% survival on ciprofloxacin and 21% survival on ampicillin. This shows also that the inhibition of either transcription or translation can lead to a response that protects against other antibiotics.

### 5.7. Environment and growth state-dependent toxins

An exact understanding of how persister cell heterogeneity arises is still lacking, but there is evidence pointing to a contribution of TA systems. During the stationary phase, the end phase of the slow-growth rate response, the persister fraction of *E. coli* is about 1 in 100. (We note that those persister numbers are dependent on the antibiotic treatment, e.g. ciprofloxacin versus ampicillin.) *Escherichia coli* mutants lacking *hipBA* and *relBE* activities do not display stationary phase persisters, indicating their involvement in persister cell formation during the slow-growth response [3]. And, the overexpression of TisB gives rise to persisters during growth, but not in stationary phase [65]. Also, in biofilms dedicated TA systems play a role, like the dinJ/yafQ system [66]. The toxin MqsR has been linked to quorum sensing in biofilms in *E. coli*, suggesting a link between the cell density in biofilms and maintenance of stable persister fractions [93]. These examples indicate that different TA systems can induce persister formation at different growth states. So, independent of the growth rate-mediated mechanisms, condition-specific mechanisms for persister formation appear to exist, which reinforces the idea of specialized persister types and how this might be achieved.

The importance of persister cell heterogeneity and its generation by different TA systems was shown by Norton & Mulvey [94]. They show that the TA systems needed for stress tolerance of a pathogenic *E. coli* strain in the kidney are different from those needed in the bladder. This suggests that needed persister adaptations vary across harsh environments, which requires alternative TA systems, and leading to a persister cell heterogeneity that is condition dependent. This concept is in agreement with the findings of Pandey & Gerdes [95]; they found that the TA systems of free-living prokaryotes are different from those of restricted and obligate intracellular organisms, by comparing 126 sequenced and annotated bacterial genomes.

## 6. Conclusion

Whereas the regulation of some responsive systems such as two-component signalling networks or operons regulated by several transcription factors can be understood by focusing on them in isolation, this may not be so simple for the formation of persister cells. The mechanisms for persister cell formation appear deeply intertwined with key regulatory processes in microbial physiology, related to growth and survival, both key determinants of long-term fitness.

It is likely that persister cell formation is a process associated with the onset of slow growth, regardless of whether this period will be transient or prolonged, or whether this is achieved by the administration of an antibiotic, a nutrient transition, a nutrient depletion (stationary phase) or the activation of a toxin. This suggests that the main mechanisms for persister cell formation are responsive. Chance-based persister cell formation does nonetheless appear to occur also. It is probably mostly relevant during conditions of fast growth and involves improbable random events such as cells experiencing an unlikely nutrient depletion (a key enzyme is expressed below a threshold level) or entering a state in which one of its toxin gets activated by chance.

Bistability and random phenotypic switching are both needed for the creation and maintenance of stable subpopulations in an isogenic population of cells. It appears that the regulatory network associated with growth and stress tolerance, involving ppGpp, sigma factor competition and the regulatory roles of *σ*^S^, indeed involves several positive feedback loops that can give rise to bistability and influence the activation of particular TA systems. That two subpopulations are formed, one growing and one stress tolerant, has probably to do with a fundamental trade-off between growth and stress tolerance, due to finite-biosynthetic resources, which prevents cell from growing fast and being highly stress tolerant at the same time.

Quantitative aspects of persister cell formation, such as the precise fractions of two subpopulations and their lifetime, have been rationalized with several theoretical approaches. These approaches give some rules of thumbs, for instance that the persister cell fraction probably resembles the chance for the event that kills all the growing cells and the ‘dormancy’ lifetime of the persister cell resembles the duration of such an event. Although such conclusions were drawn from models that may be overly simplified, they provide useful insights to keep in mind when persister cells from different microbial species are compared, as their differences are due to niche differences that have selected parameter settings for the persister cell formation mechanisms.

Although inductive reasoning has its drawbacks, we have the impression that the basics of persister cell formation, e.g. it being related to growth versus stress regulation, it being responsive to the induction of slow growth, it being dependent of bistability and random phenotype switching, it involving TA systems, is evolutionarily conserved across microbial species. This therefore allows for a single-species-overarching conceptual framework for understanding of persister cell formation. We hope that this review contributed to this.

We end by saying that microbial species are not all that different at the molecular level, despite the enormous microbial species biodiversity. This may explain why microbial species are so similar when one looks at them from a systems-biological or physiological perspective. For instance, their genome organization and gene processes obey the same principles; their proteins obey the same physical biochemistry, including kinetics and diffusion properties; their metabolism makes the same precursor molecules, including energy equivalents, that drive the biosynthesis of the same macromolecular building blocks (i.e. proteins, membranes, and DNA and RNA); their evolutionary trade-offs are probably very similar too, given that they all suffer from finite biosynthetic resources, associated trade-offs, and that evolutionary success is measured with the same long-term fitness measure (time-averaged growth rate). These similarities make conceptual approaches that span the associated scales of molecular biology, physiology and fitness possible. We hope that many of such integrative biology endeavours will follow and contribute to our search for unity in microbiology.

## Data Availability

The data are provided in electronic supplementary material [96].
